# The Anti-Inflammatory Effect of Feiyangchangweiyan Capsule and Its Main Components on Pelvic Inflammatory Disease in Rats via the Regulation of the NF-*κ*B and BAX/BCL-2 Pathway

**DOI:** 10.1155/2019/9585727

**Published:** 2019-06-13

**Authors:** Yao Li, Qian Yang, Zhi-hui Shi, Min Zhou, Li Yan, Hua Li, Yan-hua Xie, Si-wang Wang

**Affiliations:** ^1^The College of Life Sciences, Northwest University, Xi'an, 710069, China; ^2^Department of Natural Medicine, School of Pharmacy, The Fourth Military Medical University, Xi'an, 710032, China; ^3^Shaanxi Junbisha Pharmaceutical Limited Company, Xianyang, 712000, China; ^4^School of Pharmacy, Shaanxi University of Chinese Medicine, Xianyang 712000, China

## Abstract

Although gastroenteritis and pelvic inflammatory disease (PID) occur in the gastrointestinal tract and pelvis, respectively, they display similar pathogeneses. The incidence of inflammation in these conditions is usually associated with dysbacteriosis, and, at times, they are caused by the same pathogenic bacteria,* Escherichia coli* and* Streptococcus aureus*. Feiyangchangweiyan capsule (FYC) is a traditional Chinese patent medicine that is widely used to treat bacterial dysentery and acute and chronic gastroenteritis. However, whether it has an effect on PID is unclear. The aim of this study was to investigate the anti-inflammatory effect of FYC and its main components, gallic acid (GA), ellagic acid (EA), and syringin (SY), on a pathogen-induced PID model and illustrate their potential mechanism of action. Female specific pathogen-free SD rats (n = 1110) were randomly divided into control, PID, FYC, GA, EA, SY, GA + EA, GA + SY, EA + SY, GA + EA + SY, and Fuke Qianjin capsule (FKC) positive groups. Histological examination and enzyme-linked immunosorbent assay (ELISA) were carried out as well as western blot analysis to detect the expression of NF-*κ*B, BAX, BCL-2, and JNK. In this study, FYC and its main components dramatically suppressed the infiltration of inflammatory cells, reduced the production of IL-1*β*, TNF-*α*, and MCP-1, and elevated the IL-10 level to varying degrees. We also found that FYC and its main components inhibited the expression of BAX induced by infection and increased the expression of Bcl-2. FYC, GA, EA, and SY could also block the activation of the NF-*κ*B pathway. Finally, we found that the phosphorylation of JNK could be decreased by FYC, GA, and SY. FYC and its main components exhibit anti-inflammatory effect on a pathogen-induced PID model by regulating the NF-*κ*B and apoptosis signaling pathways.

## 1. Introduction

Pelvic inflammatory disease (PID) is the most frequently observed disorder of the female upper genital tract due to pathogens [1]. Many pathogenic microorganisms have been implicated in this disorder including the sexually transmitted bacteria,* Neisseria gonorrhoeae* and* Chlamydia trachomatis*, and the increase in vaginal flora such as* Gardnerella vaginalis*,* Streptococcus aureus,* and* Escherichia coli* [2, 3]. There are no evident initial PID symptoms in patients; however, as time progresses, the disorder becomes recurrent or chronic, and its sequelae of infertility, ectopic pregnancy, and chronic pelvic pain serve as important public health issues [4, 5]. The consequences of PID can be severe and treatment delay may contribute to the chance of infertility [6, 7]. Antibiotic therapies are the first choice of treatment for PID, but when used on a regular basis, they increase the likelihood of antibiotic resistance. Therefore, it is necessary to develop new complementary and alternative medicine to treat PID.

Feiyangchangweiyan capsule (FYC), a traditional Chinese medicine (TCM), is composed of* Euphorbia hirta *L.,* Polygonum chinense *L., and* Ilex rotunda *Thunb. This traditional Chinese patent medicine was approved by China's Food and Drug Administration (CFDA) and is manufactured by Shaanxi Junbisha Pharmaceutical Limited Company. FYC is widely used to treat bacterial dysentery, acute gastroenteritis, and chronic gastroenteritis [8]. However, whether it has an effect on PID is unclear, and its active ingredients and the mechanisms it employs to treat gastrointestinal inflammation remain unknown. Monarch, minister, assistant, and guide play an important role in TCM prescription but the anti-inflammatory effect of FYC is prioritized when considering its therapeutic properties and functions.

*E. hirta* belongs to the plant family Euphorbiaceae and genus* Euphorbia* [9]. Its ethanolic and aqueous extracts have been reported to inhibit the growth of organisms, such as* E. coli*,* Staphylococcus aureus*,* Pseudomonas aeruginosa*, and* Bacillus subtilis*, to varying degrees [10–13]. The extract of aerial parts of* E. hirta* displayed dose-dependent anti-inflammatory effects in the phorbol acetate-induced ear inflammation mice model [14].* P. chinense*, a perennial herb belonging to the family Polygonaceae, is widely used as a folk medicine for different purposes worldwide. In China,* P. chinense* is commonly consumed as a treatment for diarrhea and enteritis [15]. Furthermore, it is used to treat inflammation of the female genital tract in TCM clinical practice [16].* I. rotunda* belongs to the family Aquifoliaceae and has been traditionally used to treat common cold, urinary tract infection, and cardiovascular disease. Its extract has been demonstrated to display anti-inflammatory and antioxidative effects [17, 18].

**Figure 1:**
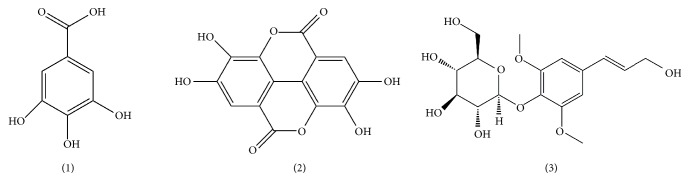
Chemical structures of gallic acid (1), ellagic acid (2), and syringin (3).

FYC contains a variety of components, but its anti-inflammatory and antibacterial activity can be attributed to the following elements: flavonoids, phenolic acids, ascorbic acid, etc. [19]. Our previous studies demonstrated that the main effective components of FYC are gallic acid (GA** 1**, Figure 1), ellagic acid (EA** 2**, Figure 1), and syringin (SY** 3**, Figure 1) [19]. Microbial components have been shown to be involved in the pathogenesis of enterogastritis as well as PID. To add, the interaction between gastrointestinal microbiota and vaginal flora has been widely confirmed to impact this process. Therefore, based on the above theories and our preresearch data of the antibacterial effect of FYC, we endeavored to investigate the pharmacological effect and mechanism of FYC and its principal components on PID in rats.

## 2. Materials and Methods

### 2.1. Materials

*E. coli* (ATCC25922) and* S. aureus* (ATCC25923) were purchased from American Type Culture Collection (Manassas, VA, USA). Enzyme-linked immunosorbent assay (ELISA) kits for interleukin-1*β* (IL-1*β*), tumor necrosis factor-*α* (TNF-*α*), interleukin-10 (IL-10), monocyte chemotactic protein-1 (MCP-1), and caspase-3 were obtained from MultiSciences (Hangzhou, China). Nuclear factor *κ*B (NF-*κ*B), I*κ*B, BCL-2, BAX, JNK, and *β*-actin antibodies were purchased from Cell Signaling Technologies (Danvers, MA, USA). FYC was provided by Shaanxi Junbisha Pharmaceutical Co. Ltd. (Xianyang, China). Fuke Qianjin capsule (FKC) was purchased from Zhuzhou Qianjin Pharmaceutical Co. Ltd. (Hunan, China). GA (C_7_H_6_O_5_, FW = 170.12, purity ≥99.9%; the main active constituent of* E. hirta*), EA (C_14_H_6_O_8_, FW = 302.19, purity ≥98.8%; the main active constituent of* P. chinense*), and SY (C_17_H_24_O_9_, FW = 372.37, purity ≥98.5%; the main active constituent of* I. rotunda*) were purchased from Chengdu Biopurify Phytochemicals Ltd. (Chengdu, China).

### 2.2. Rat PID Model Construction and Sample Collection

Female specific pathogen-free SD rats (weight, 180–220 g; age, 8-week-old) were obtained from the Experimental Animal Center of the Fourth Military Medical University. The experimental protocols were approved by the Laboratory Animal Center of the Academy of the Fourth Military Medical University. The animals were housed under a 12-h dark-light cycle (light on, from 7:00 to 19:00) under a temperature of 22–24°C and relative humidity of 60–65%. All rats were randomly divided into the control, PID, FYC (1.2 g/kg), GA (210 mg/kg), EA (30 mg/kg), SY (35 mg/kg), GA (105 mg/kg) + EA (15 mg/kg), GA (105 mg/kg) + SY (18 mg/kg), EA (15 mg/kg) + SY (18 mg/kg), GA (70 mg/kg) + EA (10 mg/kg) + SY (12 mg/kg), and FKC (2.4 g/kg) groups, each with 10 rodents. After acclimation for 7 days, the PID model was established using improved methods reported previously [20–22]. Rats were anesthetized intraperitoneally with 30 mg/kg of pentobarbital. The lower abdomen was shaved and swabbed with Betadine. A 1-cm incision was made ventrally in the skin, and the peritoneum was directly and bluntly dissected over the cervix. Microbe-mixing solution with* E. coli* (1 × 10^8^ CFU/mL) and* S. aureus* (1 × 10^8^ CFU/mL) was prepared, uterine horns were exposed, and 50 *μ*L of microbe-mixing solution was immediately injected into the uteri proximal to the distal of the branching point in the direction of the endocervix by use of an insulin syringe fitted with a 30G needle. The skin was then closed with a standard surgical staple. Control group rats were inoculated intracervically with sterile saline. After 24 h of infection, each group of rats was orally administered the corresponding drugs. After 14 days, rats were sacrificed by cervical dislocation, and blood samples and uterus were collected and stored at −80°C for further evaluations. Meanwhile, a fraction of the uterus was immersed in neutral-buffered formalin (10%) for hematoxylin-eosin (H&E) staining.

### 2.3. Evaluation of Uterus Appearance

A researcher, blinded to the grouping, scored the degree of inflammation in rat uterus based on the apparent expression. The appearance of the uterus included edema, hyperemia, and white pus within the uterine cavity. Based on different intensities, each feature was counted on a three-point scale: 0 (not present), 1 (moderately visible), and 2 (severe). The weight of the uterus was then taken and used to calculate the uterine index (weight of horn/body weight of rat × 100).

### 2.4. Histological Evaluation

For histological evaluation, the uterus was cut into 2-*μ*m sections and stained with hematoxylin and eosin. Three parts of each slide (tissue) were evaluated under low-power microscopy field (×200). The extent of inflammatory cell infiltration was evaluated by the semiconducted method (grade, 0 to 3).

### 2.5. Enzyme-Linked Immunosorbent Assay (ELISA)

The amounts of IL-1*β*, TNF-*α*, IL-10, and MCP-1 in serum were determined with ELISA kits (MultiSciences). The concentrations of cytokines and chemokines were expressed as pg/mL protein of homogenate.

### 2.6. Western Blot Analysis

Tissue samples of the upper genital tract were homogenized and lysed in ice-cold RIPA lysis buffer, then mixed with 2× SDS-PAGE sample buffer, boiled for 10 min, and then resolved by 10% SDS-PAGE. The proteins were then transferred onto polyvinylidene difluoride (Millipore, Billerica, MA, USA) membranes, blocked at 37°C for 60 min with 5% nonfat dry milk, and then reacted with properly diluted monoclonal antibodies (1:1000) including NF-*κ*B p65, p-NF-*κ*B p65, p-I*κ*B*α*, B*α*, BAX, BCL-2, JNK, and p-JNK. After washing, the membranes were incubated with peroxidase-linked goat anti-rabbit IgG secondary antibody (1:1000; Santa Cruz Biotechnology) at 37°C for 1 h. Protein bands were detected using horseradish peroxidase-conjugated goat anti-mouse IgG antibodies followed by an enhanced chemiluminescence reaction (Pierce Biotechnology, USA).

### 2.7. Statistical Analyses

All data are presented as mean ± standard deviation (SD). The differences between two datasets were evaluated using Student's* t*-test with SPSS 18.0 statistical software (SPSS, USA). One-way analysis of variance (ANOVA) was used to compare the difference between more than two datasets. Difference was considered statistically significant when the p value was <0.05.

## 3. Results

### 3.1. Uterine Appearance and Histopathological Changes

**Figure 2:**
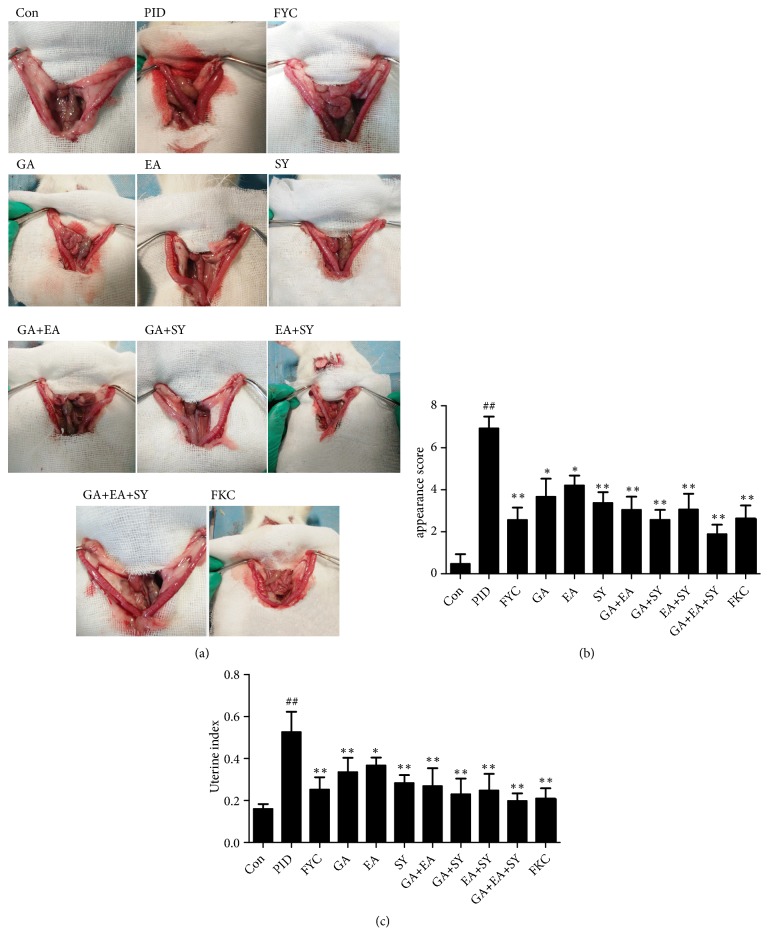
Effect of FYC and its main components on pathogen-induced inflammation of the uterus. (a) Appearance of the uterus in the different groups. White pus within the uterine cavity is indicated as ↑. (b) Semiquantitative scores of the inflammation for the appearance of uterus. (c) Index of the uterus for each group. Each bar represents the mean ± SD (##*P* < 0.01 vs. control group, *∗P* < 0.05, *∗∗P *< 0.01 vs. PID group).

To assess the anti-inflammatory activity of the main components of FYC, uterine appearance was scored as shown in Figure 2. We found no obvious changes in the uterine horns in the control group; however, in the PID group, the uteri showed different degrees of lesion with uterine enlargement, hyperemia, white pus within the uterine cavity, and uterine wall thickening. The uterine index (weight of horn/body weight of rat × 100) of the PID group was much higher than that of the control group. However, these lesions were significantly attenuated by the administration of FYC and its main components, and the scores for the inflammation of uterine appearance showed dramatic differences between the PID and treatment groups.

**Figure 3:**
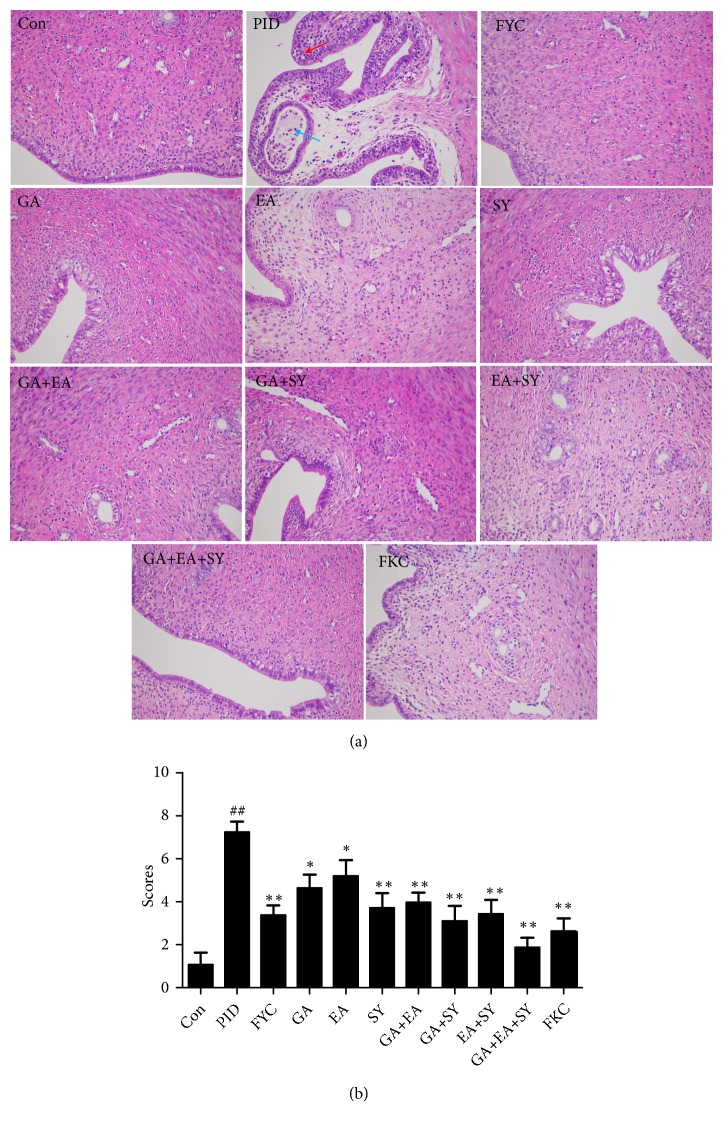
Effect of FYC and its main components on the pathogen-induced infiltrations of inflammatory cells in the upper genital tract. (b) Typical micrographs (×200) of the uterus stained with H&E. Representative inflammatory cells are indicated as ↑. (b) Semiquantitative scores of the infiltration of inflammatory cells. Each bar represents the mean ± SD (##*P *< 0.01 vs. control group, *∗P* < 0.05, *∗∗P* < 0.01 vs. PID group).

The results of the histological assessment performed with a light microscope are presented in Figure 3. In the control group, the luminal epithelium was integrated into the endometrium, the glands were enlarged, and the smooth muscles were arranged regularly with no degeneration. As shown in Figure 3, the uteri from the PID group exhibited severe pathological changes, (e.g., hyperemia, hemorrhage, and the shedding of epithelial cells). Moreover, the uterine tissues were infiltrated by masses of inflammatory cells, most of which were polymorphonuclear (PMN) cells. These results demonstrate the occurrence of inflammation in the upper genital tract. Nevertheless, compared to the PID group, histopathological changes and the infiltration of inflammatory cells in the drug intervention groups were significantly decreased in the upper genital tract. Semiquantitative results also showed notable differences in the histopathological changes between the PID group and medicated groups.

### 3.2. Effect of FYC and Its Main Components on the Excessive Production of Cytokines and Chemokines

**Figure 4:**
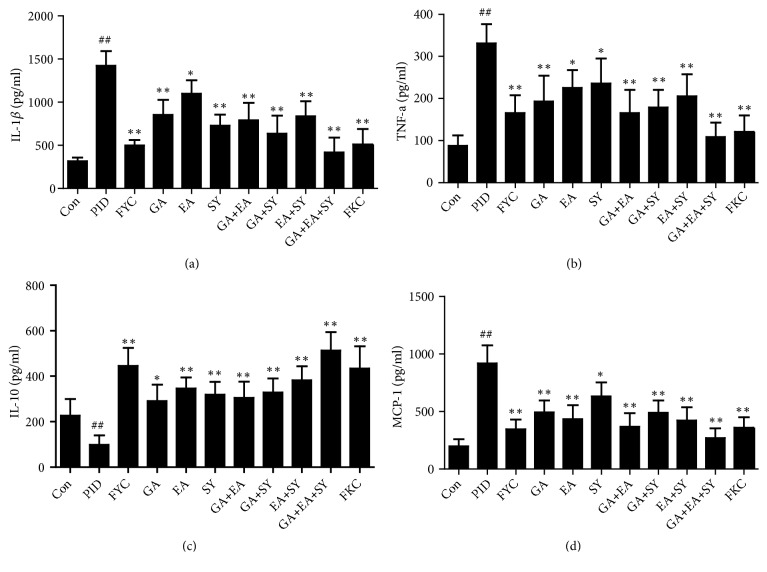
Effect of FYC and its main components on the pathogen-induced over-production of IL-1*β*, TNF-*α*, and MCP-1 and the reduction of IL-10 in the upper genital tract. Each bar represents mean ± SD (##*P* < 0.01 vs. control group, *∗P *< 0.05, *∗∗P* < 0.01 vs. PID group).

Inflammatory response and inflammatory cell infiltration are related to the excessive production of cytokines and chemokines. In this research, we used ELISA kits to measure IL-1*β*, TNF-*α*, IL-10, and MCP-1 in the serum of rats. As shown in Figure 4, IL-1*β*, TNF-*α*, and MCP-1 levels were dramatically increased in the PID group compared to the control group. Meanwhile, IL-10 was sharply decreased in the PID group compared to the control group. After oral administration of FYC and its main components, the pathogen-induced production of IL-1*β*, TNF-*α*, and MCP-1 was reduced by varying degrees. In addition, IL-10 in the serum from drug intervention groups was increased by varying degrees.

### 3.3. Effect of FYC and Its Main Components on Apoptosis in Response to PID

**Figure 5:**
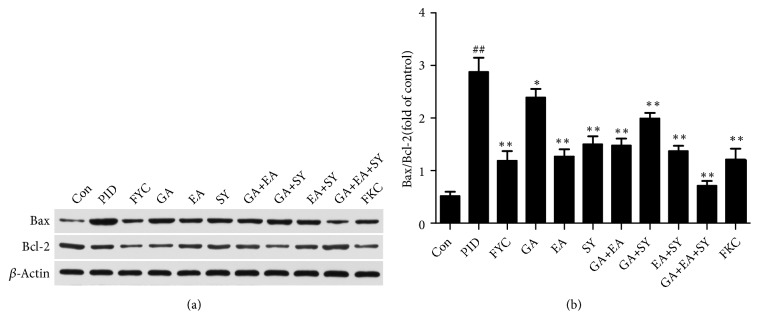
Effect of FYC and its main components on the pathogen-induced over-production of BAX/BCL-2 in the upper genital tract. Each bar represents the mean ± SD (##*P *< 0.01 vs. control group, *∗P* < 0.05, *∗∗P* < 0.01 vs. PID group).

Considering that the proapoptotic factors (BAX) and antiapoptotic genes (BCL-2) are largely associated with the progression of apoptosis, the expression levels of these apoptosis-related proteins were analyzed to investigate the mechanism of FYC and its main components in the upper genital tract. As shown in Figure 5, the level of BAX was significantly increased in the PID group whereas that of BCL-2 was decreased. After oral administration of FYC and its main components, the expression level of BAX was largely reduced and BCL-2 was increased. These results illustrate that apoptosis can be induced by bacterial infection, and FYC and its main components have an effect on apoptosis.

### 3.4. Effect of FYC and Its Main Components on NF-*κ*B Activation and JNK Expression

**Figure 6:**
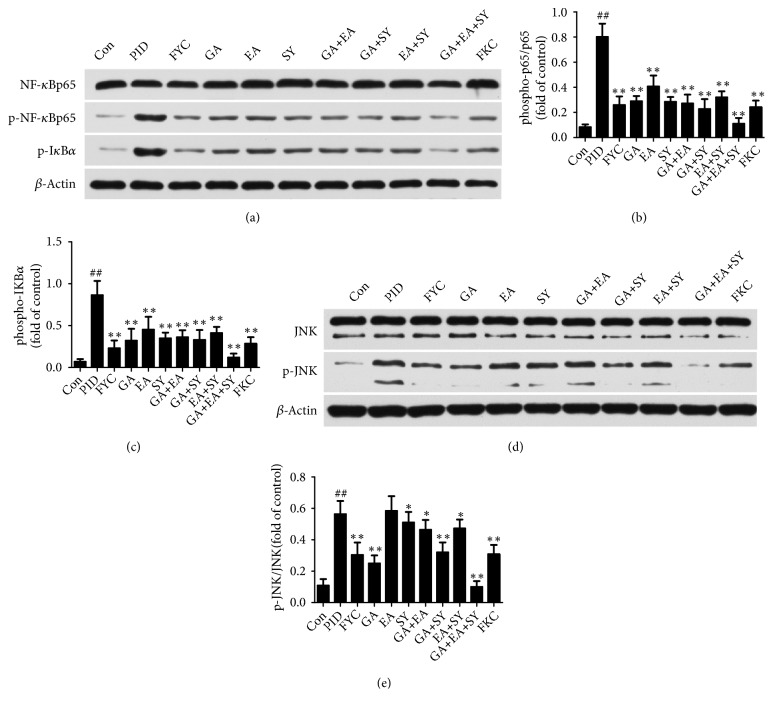
Effect of FYC and its main components on the pathogen-induced over-production of p-NF-*κ*B and p-JNK in the upper genital tract. Each bar represents the mean ± SD (##*P* < 0.01 vs. control group, *∗P* < 0.05, *∗∗P *< 0.01 vs. PID group).

In the uterus, the NF-*κ*B pathway plays a pivotal role in inflammatory response. Hence, to investigate the underlying mechanism of the anti-inflammatory effects of FYC and its main components, NF-*κ*B p65, p-NF-*κ*B p65, and p-I*κ*B*α* and the protein level of upstream effectors of the NF-kB signaling pathway (p-JNK/JNK) were detected by western blot analysis. As shown in Figure 6, compared to the control group, the expression of p-NF-*κ*B p65, p-I*κ*B*α*, and p-JNK was significantly increased in the PID group but was alleviated to varying degrees by treatment with FYC, GA, and SY.

## 4. Discussion

PID is a common disease in women and is referred to as inflammation of the upper genital tract including the ovaries, fallopian tubes, and their surrounding structures [23]. It is widely believed that* C. trachomatis* and* N. gonorrhoeae* are the main pathogens associated with PID, but* C. trachomatis* or* N. gonorrhoeae* infection may also permit the entry of opportunistic pathogen (such as* Mycoplasma hominis* and other anaerobes) into the upper genital tract [24, 25]. PID is a common cause of morbidity in young women. In the UK and the US, approximately 4% to 12% of young women are infected with chlamydia [26], 5% to 30% of whom are at risk of developing PID, if left untreated [27]. Of these PID patients, 10% to 20% will become infertile, 40% will develop chronic pelvic pain, and 10% who conceive will have an ectopic pregnancy [28]. In China, pathogenic bacteria causing PID also include* E. coli* and* S. aureus*. This phenomenon of pathogenic bacterial difference may akin to the living habits and environment in each country. Therefore, we attempted to use* E. coli* and* S. aureus* to provoke an augmented inflammation in the upper genital tract to construct the model used.

According to microecology, an organism is a large microecosystem. Vaginal flora and intestinal flora are the two main components of the human body's microecosystem and the balance of the two is interdependent as stated in the TCM theory [29]. Based on the literature search, every traditional Chinese medicine has antibacterial and anti-inflammatory effects; therefore, we assumed that FYC may also have a therapeutic effect on PID and this hypothesis was verified in our previous experiments. To investigate the potential mechanism of the main component of FYC and illustrate the multitarget effect of TCM, we selected GA, EA, and SY, which are regarded as the main components of FYC in our previous pharmacokinetic study, to treat rats with PID.

Neutrophils often appear at the sites of inflammation in different pathogen-infected tissues. When a mass of neutrophils reach the infection site of a tissue, they will release many inflammatory factors, oxygen free radicals, and proteolytic enzymes to kill the pathogens. However, excessive products will also induce tissue damage and lead to structural disease in the upper genital tract. Patton et al. [30] reported that tissue damage including epithelial cell degeneration occurs in close approximation to lymphocytes and, in our study, many neutrophils and lymphocytes infiltrated into the epithelium of the upper genital tract of rats with PID, and each therapeutic group showed a better effect on attenuating the infiltration of inflammatory cells and alleviating this tissue damage.

Toll-like receptors (TLRs) are typically expressed in epithelial cells and mediate the innate immune system [31]. After pathogen infection and recognition of immunogens by local TLRs, the innate and adaptive immune systems are stimulated to fight against infection. Compared to the adaptive immune system, the innate immune system initiates more quickly and efficiently responds to the infection via surface defenses, cytokine elaboration, complement activation, and phagocytic responses [32, 33]. The pro- or anti-inflammatory cytokines produced by the host's immune system play essential roles in the inflammatory response. For instance, IL-1*β*, IL-10, MCP-1, neutrophils, or macrophages serve protective or destructive roles in PID [34, 35] and, in our study, cytokines and chemokines involving IL-1*β*, TNF-*α*, and MCP-1 were increased, whereas IL-10 was degraded in the uterus of rats with PID. After an oral administration of FYC, GA, EA, SY, and any two or three random combinations of GA, EA, and SY, respectively, IL-1*β*, TNF-*α*, and MCP-1 were significantly decreased whereas IL-10 was increased in a different manner. Furthermore, the effects of the GA + EA + SY group were better than the GA + EA, GA + SY, and EA + SY groups and were superior to the GA, EA, and SY groups.

Apoptosis is perceived as an important part of various processes involving normal cell turnover, hormone-dependent atrophy, and chemical-induced cell death [20]. However, inappropriate apoptosis may aggravate damage caused by some diseases, such as inflammation, tumor, or other human neurodegenerative diseases. Necrosis is commonly induced by aggressive inflammatory response, inappropriate apoptosis, and highly regulated genetic and biochemical processes [36] whereas apoptosis plays a critical role in the outcome of pathogenic infections [37]. The BCL-2 family is most notable for its regulation of apoptosis and the consistent proteins from this family can promote or inhibit apoptosis, such as the proapoptotic BAX and the antiapoptotic BCL-2 [38, 39]. Compared to the control group, the expression of BAX on the pelvic of rats with inflammation was found to increase and BCL-2 expression was dramatically suppressed. Administering FYC and its main components reversed these changes, especially EA and SY treatment, and the function of the combination group was significantly superior to that of the monomer groups.

**Figure 7:**
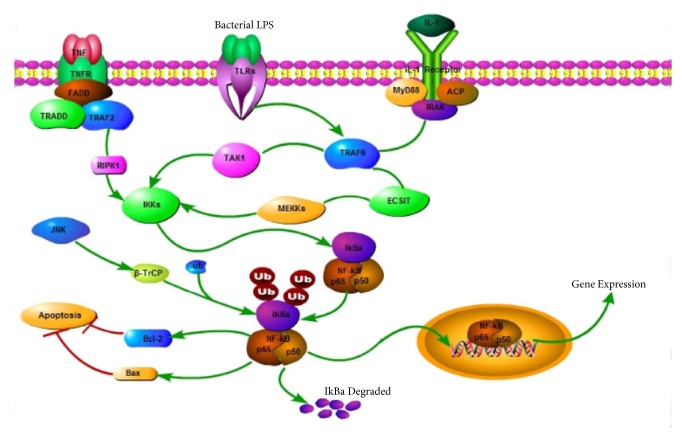
Signaling pathway.

The classic NF-*κ*B pathway can be activated by the IRAK complex during inflammation or an immune response in an organism. NF-*κ*B is a transcription factor retained in the cytoplasm when bound to I*κ*B family members without any activity [40, 41]. Based on a report, an infection of the uterus caused by pathogens results in the phosphorylation of the I*κ*B family and leads to ubiquitination and subsequent degradation. I*κ*B degradation results in the translocation of NF-*κ*B dimers to the nucleus and promotes the expression of inflammatory mediators, such as IL-1*β* and IL-6 [42–44]. These inflammatory mediators act as a positive feedback for further activation of NF-*κ*B, and they result in a subsequent production of more proinflammatory mediators [45]. Several pathogens such as* E. coli* and* M. pneumonia* could induce the activation of the NF-*κ*B pathway [46–49] and, according to some reports, MAPK, especially c-Jun NH (2)-terminal kinase (JNK) as the upstream kinase in the NF-*κ*B signaling pathway, can induce the activation of NF-*κ*B under some conditions [50]. JNK and p38 MAPK were induced and activated by a variety of cellular stresses, such as inflammatory cytokines [51], and their activation contributes to cell apoptosis and death [52]. Some researchers found that viral infections can induce the activation of JNK and p38 MAPK and cause injury to patients [53]. As shown in Figure 7 and as demonstrated in our previous research, JNK phosphorylation was decreased by FYC. Our results also showed that a mixture of bacterial infections activated the NF-*κ*B pathway in the uterus of rats with PID; however, following an oral administration of FYC, GA, EA, and SY, the expression of p-NF-*κ*B and p-I*κ*B was suppressed. p-JNK was also found to be significantly reduced by FYC and GA. Generally, FYC and its main components may exert their anti-inflammatory effects by blocking the activation of the NF-*κ*B pathway and regulating the apoptosis signaling pathway. To add, the anti-inflammatory effect of the compatibility group (GA + EA + SY) was superior to the FYC group; however, this difference may be due to the restriction of the FYC dose administered. The phytochemical characteristics of traditional Chinese medicines and their compatibility application are clearly worthy of further investigation.

## 5. Conclusions

FYC and its main components (GA, EA, and SY) showed dramatic anti-inflammatory activity in rats with pathogen-induced PID, especially in the group that contained the three primary components of FYC (GA + EA + SY). This led to the inhibition of neutrophil and lymphocyte infiltration and a reduction in the excessive production of cytokines or chemokines. The active ingredients of FYC (GA, EA, and SY) were demonstrated to be bioactive components, providing the theory basis for its compatibility as a TCM. Finally, the potential mechanism of this effect was revealed to be related to the regulation of the NF-*κ*B and apoptosis pathways.

## Data Availability

All data about this research are included in the manuscript.
